# Modeling of Disordered Protein Structures Using Monte Carlo Simulations and Knowledge-Based Statistical Force Fields

**DOI:** 10.3390/ijms20030606

**Published:** 2019-01-31

**Authors:** Maciej Pawel Ciemny, Aleksandra Elzbieta Badaczewska-Dawid, Monika Pikuzinska, Andrzej Kolinski, Sebastian Kmiecik

**Affiliations:** 1 Faculty of Chemistry, Biological and Chemical Research Center, University of Warsaw, Pasteura 1, 02-093 Warsaw, Poland; maciej.ciemny@fuw.edu.pl (M.P.C.); adawid@chem.uw.edu.pl (A.E.B.-D.); m.pikuzinska@student.uw.edu.pl (M.P.); kolinski@chem.uw.edu.pl (A.K.); 2 Faculty of Physics, University of Warsaw, Pasteura 5, 02-093 Warsaw, Poland

**Keywords:** coarse-grained, CABS model, MC simulations, statistical force fields, disordered protein, protein structure

## Abstract

The description of protein disordered states is important for understanding protein folding mechanisms and their functions. In this short review, we briefly describe a simulation approach to modeling protein interactions, which involve disordered peptide partners or intrinsically disordered protein regions, and unfolded states of globular proteins. It is based on the CABS coarse-grained protein model that uses a Monte Carlo (MC) sampling scheme and a knowledge-based statistical force field. We review several case studies showing that description of protein disordered states resulting from CABS simulations is consistent with experimental data. The case studies comprise investigations of protein–peptide binding and protein folding processes. The CABS model has been recently made available as the simulation engine of multiscale modeling tools enabling studies of protein–peptide docking and protein flexibility. Those tools offer customization of the modeling process, driving the conformational search using distance restraints, reconstruction of selected models to all-atom resolution, and simulation of large protein systems in a reasonable computational time. Therefore, CABS can be combined in integrative modeling pipelines incorporating experimental data and other modeling tools of various resolution.

## 1. Introduction

There is a growing body of evidence that some proteins act in multiple structural states [1]. It has been demonstrated that the ability of these proteins to switch between distinct structural states may be crucial for their function and regulation [1]. Additionally, a number of key biological functions have been proven to be performed by disordered or partially unstructured proteins [2]. Some proteins fold and obtain their structure only upon binding to their partners, while others form so called “fuzzy complexes” in which both proteins retain a certain degree of disorder [3]. These discoveries modified the core biochemistry principle of “structure determines function”. As for now, a consensus has been reached that protein function may be a result of an interplay between protein structure and its dynamics [4,5].

Internal protein motions may be studied both experimentally and with computational methods [6,7]. For example, nuclear magnetic resonance (NMR) spectroscopy is one of the richest sources of information on protein structure and dynamics, especially when accompanied with assisting methods that enhance resolution or provide an additional insight into the dynamics of structures [8]. This approach, however, results in an averaged image of the structural ensemble.

A variety of computational techniques have been developed to assist these challenging experimental studies [7,9]. In the last decades, molecular modeling was dominated by structure-based models or Go-like models (approaches that are biased toward known folded conformations [10,11]). These indeed lead to significant speedup of simulations but may result for example in an unrealistic picture of protein folding, which in reality may also depend on non-native interactions [12,13,14]. 

Recent works show that methods combining experimental data and computational approaches may produce the most promising pictures of protein equilibrium dynamics [15,16]. However, the development of these methods poses a number of challenges—both in terms of the validity of the approach and its computationally efficient implementation [17].

Molecular dynamics (MD) has been so far the most widespread computational method for the investigation of protein motions [18]. However, standard all-atom MD implementations are limited to sub-microsecond timescales and may suffer from limited sampling despite recent significant advances in code optimization and hardware [19]. To overcome this problem various MD extensions have been proposed. These extensions include for example replica-exchange MD, meta-dynamics, Markov state models and simulated annealing algorithms [6,20,21,22,23]. 

A number of non-MD sampling methods have also been developed to provide a comprehensive image of protein dynamics using limited computational resources. Of these, Monte Carlo (MC) is perhaps the most commonly used and generally applicable sampling method [11]. Monte Carlo randomly generates conformations and uses an energy-based acceptance criterion that promotes pseudo-trajectory convergence to an energetic minimum. On the expense of losing a direct image of the timescales or kinetics of the ensemble, MC manages to overcome some of the major limitations of MD [24]. 

Aside from the sampling method, a further extension of effective timescales is possible by using a simplified representation of protein structures to reduce the number of a system’s degrees of freedom. The accuracy of the available coarse-grained (CG) models may vary from detailed, almost atomistic representations (Primo [25], Rosetta [26]), medium resolution models (in which a single amino acid is represented by three to five beads: UNRES [27], CABS [28], AWSEM [29], MARTINI [30], PaLaCe [31]), and Scorpion [32]) to significantly simplified models like SURPASS [33,34]. Applications and implementations of these and other CG models are described in detail in a recent review [11]. 

In addition to the representation and sampling method, the choice of the force field to perform the simulation determines the success of modeling. Traditionally, force fields are divided into two main groups: physics-based, which involve (usually pairwise) interaction terms [35], and those employing a statistical approach; however, most of the successful approaches are usually a mixture of the two. A statistical force field is constructed using the probability of a chosen observable (or a set of observables) in a given ensemble of structures [36]. Early attempts focused on straightforward pairwise contacts [37]; however, with further development, more complex observables were analyzed. This resulted in a generation of knowledge-based force fields, or scores, for various representations, coarse-grained and all-atom: CABS [28], Rosetta [38], DOPE [39], GOAP [40], QUARK [41], Bcl::Score [42] or BACH [36]. Newly developed approaches go a step further and improve the results by combining these methods with experimental data [43,44]. An example of such approach is RosettaEPR [45], which includes distance data from site-directed spin labeling electron paramagnetic resonance experiments. It is generally agreed that statistical force fields frequently allow more accurate scoring than physics-based potentials [11]. The combination of knowledge-based force fields or scores with effective sampling schemes seems to be a promising approach to a number of problems [11], such as protein structure prediction [43,44,46,47], investigation of protein interactions [48] or studies of protein dynamics [17,49,50,51].

This review briefly describes one of these approaches: an MC-based and knowledge-based interaction scheme for modeling protein–peptide interactions and unfolded states of globular proteins using the CABS coarse-grained protein model. Firstly, the main features of the CABS method will be described, with a focus on their applicability for modeling disordered or unfolded proteins or their fragments. Subsequently, representative case studies will be discussed to provide detailed insights into the modeling results obtained for systems characterized by a varying level of disorder.

## 2. CABS Dynamics and Interaction Model

Since its development, the CABS model (C-alpha, C-beta and Side chain model) has been applied to a variety of modeling problems, such as protein folding mechanisms [49,50,52,53,54,55,56,57], protein structure prediction [58,59,60,61], protein–peptide docking including large-scale conformational flexibility [62,63,64,65,66,67,68] and simulations of near-native fluctuations of globular proteins [69,70,71,72,73]. When combined with careful bioinformatics selection of the generated models, CABS proved to be one of the two most accurate structure prediction tools evaluated in the CASP (Critical Assessment of protein Structure Prediction) experiment [60]. The CABS model uses up to four atoms or pseudo-atoms per residue (see the description below), but outputs protein systems in C-alpha representation only. Therefore, for practical applications, the obtained models need to be reconstructed to all-atom representation. In various multiscale modeling tools discussed below, CABS has been integrated with the MODELLER-based reconstruction procedure [74]. Other reconstruction scenarios are also possible to ensure the best possible quality of local protein structure. This can be realized by combination of different tools for protein backbone reconstruction from the C-alpha trace and side chain reconstruction, like BBQ [75] or SCWRL [76] for example, and optionally further refinement [77].

**Figure 1:**
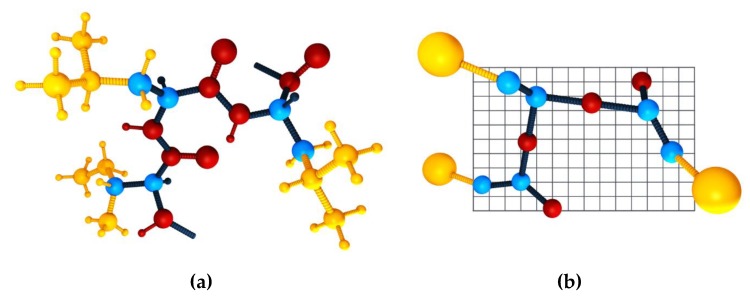
A three-residue protein fragment in: all-atom (**a**) and CABS model (**b**) representation. The spheres represent atoms: blue, C-alpha and C-beta atoms (the same in both representations); yellow, side chain atoms (one pseudo-atom in CABS); red, atoms involved in the peptide bond (one pseudo-atom in CABS placed in the geometric center of the peptide bond. A single slice (layer) of the lattice that confines the C-alpha trace in the CABS model is also presented.

In this review, we discuss the applicability of the CABS CG model and its knowledge-based statistical force field [28] to the modeling of disordered or unfolded protein states. In the CABS model the polypeptide chain representation is reduced to up to four unified atoms per residue (see Figure 1). These interaction centers represent lattice-confined C-alpha atoms, C-beta atoms, the united side chain pseudo-atom, and additionally, pseudo-atoms representing geometrical centers of peptide bonds needed to define the hydrogen pseudo-bond. An example of a polypeptide chain in CABS representation is presented in Figure 1b. Even though the restriction of the C-alpha trace to the underlying low spacing (0.61 Å [28]) cubic lattice may appear to be a drastic simplification, it is not. Allowing small fluctuations of the C-alpha, C-alpha distance enables hundreds of possible orientations of this pseudo bond, and thereby the resulting model chains do not show any noticeable directional biases. Furthermore, the averaged resolution of the C-alpha traces is acceptable and below 0.5 Å [28]. Additionally, the lattice representation enables pre-calculation of local moves and corresponding changes of interactions, leading to a few times faster simulations in comparison with otherwise equivalent continuous space CG models [11].

**Figure 2:**
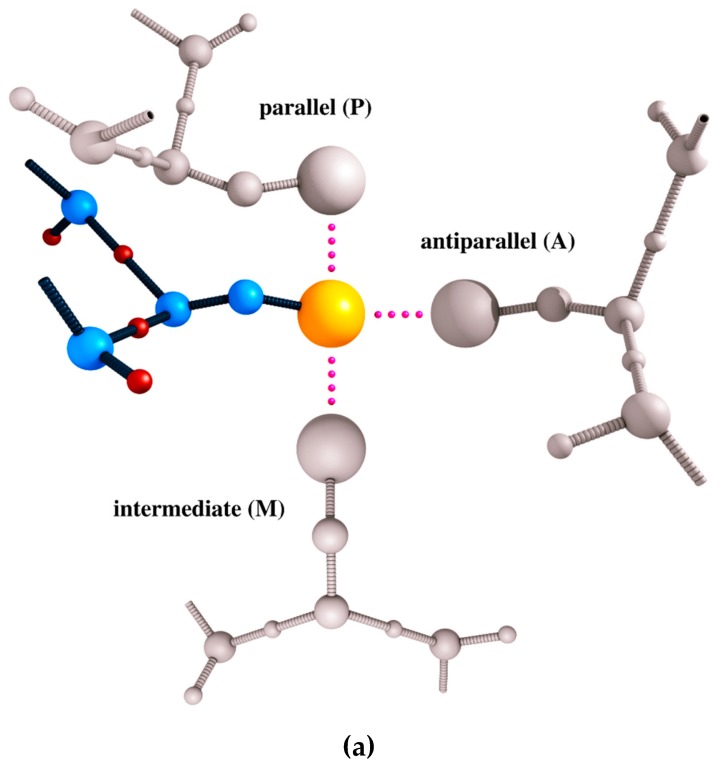

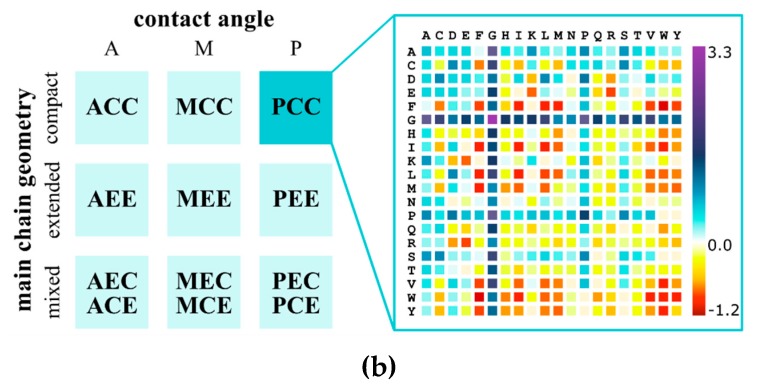
Key elements of a residue–residue interaction term in the CABS model force field. Panel (**a**) shows three examples of contact geometries in CABS representation: parallel (P), antiparallel (A), and intermediate (M), used to derive contact statistics from experimentally-derived structures of folded globular proteins. Panel (**b**) shows an example matrix of contact energies which depend on the geometry of the contacting pair, main chain geometry (compact (C) or extended (E)) for both amino acids (left part of the panel), and also on the amino acid identities (right part of the panel, the amino acids are represented using the one-letter code). The PCC matrix is presented which shows interaction energies between residues being in parallel orientation (P), where one residue belongs to a compact type of structure (C) and the second one as well (C).

The CABS model uses a knowledge-based statistical force field that consists of generic, sequence-independent interaction terms that favor protein-like conformations, and sequence-dependent interaction terms that determine some structural details [11,28,78]. The generic force field terms are derived from general features of polypeptide chains that result in protein-like behavior of the model chains. They account for properties of protein chains such as local stiffness, their biases toward secondary structures and packing compactness. The residue–residue interaction terms are derived from contact geometry statistics derived from folded globular proteins (illustrated in Figure 2a). Nevertheless, the local packing regularities in unfolded states appear to be very similar to that observed in native structures [11,28,33]. Thereby, CABS simulations provided correct pictures of protein folding [49,52,53,54,55,56,60] and flexibility of globular proteins [70,71]. 

The resulting force field takes a form of a precomputed matrix of contact pseudo-energies, presented schematically in Figure 2b. Additionally, to allow successful modeling of membrane proteins the CABS force field can be extended by introducing effective dielectric constant terms [79]. 

The main difference between CABS and other statistical force fields used in CG models of similar resolution [11] is the context and orientation dependence of side chain interaction pseudo-energy that encodes characteristic patterns observed in globular proteins. For instance, the oppositely charged side chains in single globules mostly contact in an almost parallel fashion (usually on the surface of a globule), while the antiparallel contacts (usually in the buried regions of the protein globule) are very rare. Therefore, in the context dependent force field these antiparallel contacts of oppositely charged residues are treated as repulsive. This way, the CABS force field implicitly incorporates information on the complicated interaction patterns with the solvent (via contact statistics) and its entropic contribution to system thermodynamics [11,28].

Using the mean-force force field derived from folded proteins to simulations of less-structured systems raises justified questions about the validity of this approach in studies of the disordered protein regions. The folding events observed in simulations performed using the CABS force field are consistent with both the experimental data and all-atom MD simulations [49,52,80,81]. Thus, it is hypothesized that unstructured (unfolded, partially unfolded or intrinsically disordered) proteins to a significant extent share similar stabilizing interaction patterns with the patterns observed for their well-structured counterparts [82,83].

**Figure 3:**
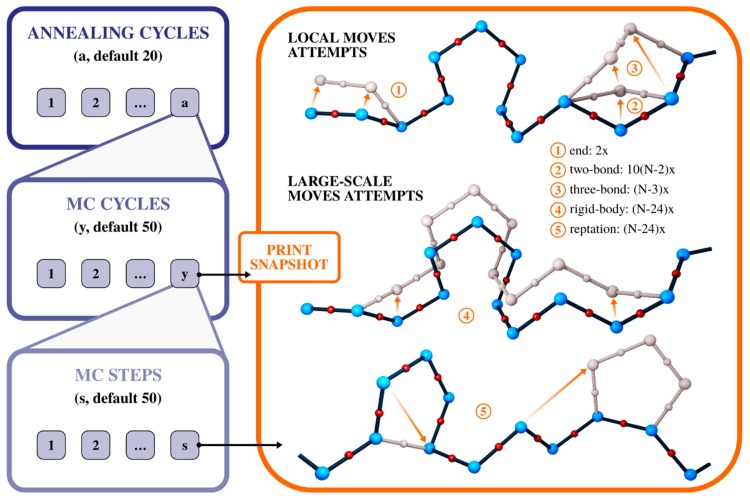
Sampling scheme of the CABS model. Blue panels show implementation details of Monte Carlo (MC) iterations (loops). The orange panel shows all motions that may be performed in a single MC step. The simulation is organized as a set of nested loops, in which the *s* number of MC steps is organized into the *y* number of cycles, and these in *a* annealing cycles (number of *a*, *y* or *s* cycles can be controlled by the user in CABS-flex and CABS-dock standalone packages [72]). In the orange panel, numbers 1 to 5 denote the available moves, presented together with the number of attempts to perform a move in each of the MC steps. The resulting trajectory is comprised of simulation snapshots saved at the end of each MC cycle.

The CABS method uses the MC asymmetric Metropolis sampling scheme that governs a set of local motions as well as multi-residue, small distance moves of the C-alpha atoms (see Figure 3). The method uses a replica exchange algorithm with simulated annealing to enhance the sampling of conformational states. The simulation is organized as a set of nested loops, in which the *s* number of MC steps are organized into the *y* number of MC cycles, and these in the *a* number of annealing cycles. Each of the MC steps consists of a per-set number of attempts to perform each of the five standard precomputed moves. The available motions and the details of implementation of the sampling scheme are presented in Figure 3.

The combination of the key features of CABS—its representation, force field and the scale of the movements used in the MC scheme—makes it suitable for the investigation of protein pseudo-dynamics. As mentioned above, the fine-grained lattice improves sampling efficiency, achieving effective timescales of milliseconds. As compared with MD, this is a considerably broader time range (in the study of flexibility of folded proteins [71] the CABS dynamics was estimated to be around 6 × 10^3^ cheaper in terms of computational cost than the classical MD). The chosen micro-motions allow (via accumulation over simulation steps) cooperative, large-scale motions. The ensemble of structures produced by the CABS method resembles a dynamic ensemble averaged over the effective timescale. Due to the nature of the method, the picture of local dynamics is distorted (on the level of local moves); however, it may be argued (based on the works mentioned above that compared our simulations with experimental data) that the long-time pseudo-dynamics recovers the realistic picture of protein motions averaged over time.

The timescale of the CABS simulations is not a priori defined and depends on the CABS simulation temperature, due to hidden entropic contributions in the force field, accounting for implicit solvent effects and multi-body interactions encoded in the statistical force field. Nevertheless, the effective timescale of MC dynamics can be approximately identified by comparison with MD trajectories from sufficiently long simulations. This comparison was thoroughly discussed previously, and the results were compared to MD results [69] and NMR ensembles [71].

The CABS model is presently used as a simulation engine of a few multiscale modeling tools that merge CABS with models reconstruction to all-atom resolution. Those include the CABS-dock method for flexible protein-peptide docking (available as a web server [62] at http://biocomp.chem.uw.edu.pl/CABSdock and a standalone application [84] at https://bitbucket.org/lcbio/cabsdock/) (accessed on 30 January 2019). In comparison to other protein–peptide docking tools, reviewed recently [85], CABS-dock offers a unique opportunity for modeling large-scale rearrangements of protein receptor structure during on-the-fly docking of fully flexible peptides. Another CABS-based tool, CABS-flex, enables fast simulations of protein flexibility (available as a web server [73] at http://biocomp.chem.uw.edu.pl/CABSflex and a standalone application [72] at https://bitbucket.org/lcbio/cabsflex/, accessed on 30 January 2019). This approach has been also incorporated as the module in the Aggrescan3D method for prediction of protein aggregation properties (available as a web server [86] at http://biocomp.chem.uw.edu.pl/A3D and a standalone application at https://bitbucket.org/lcbio/aggrescan3D, accessed on 30 January 2019). By using CABS-flex predictions, Aggrescan3D enables predicting the impact of protein conformational fluctuations on aggregation properties. Finally, the CABS model is used in the CABS-fold method for protein structure prediction: in the de novo fashion (from an amino acid sequence only), guided by user-provided templates or user-provided distance restraints (available as a web server [58] at http://biocomp.chem.uw.edu.pl/CABSfold/, accessed on 30 January 2019). The access to CABS-based tools, together with the tools description, is also available from websites of the laboratories: http://biocomp.chem.uw.edu.pl/ and http://lcbio.pl/ (accessed on 30 January 2019).

## 3. CABS Applications to Simulation of Disordered or Unfolded Proteins

**Figure 4:**
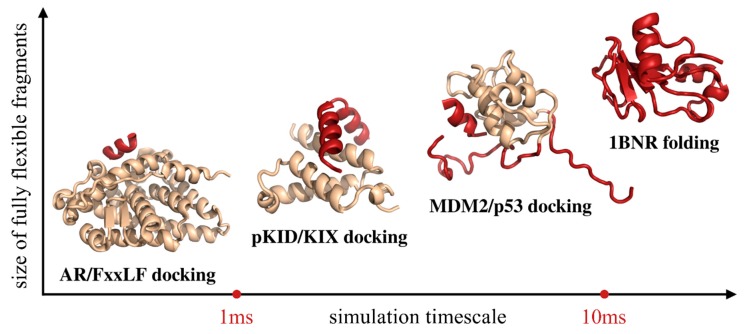
Presentation of the modeling cases discussed in this work. The modeled systems are arranged according to the size of the fully flexible fragment of the modeled system and the effective timescales required to observe their motions. The regions of the systems that were modeled as fully flexible are marked with red, while the regions in which backbone fluctuations were limited to 1 Å RMSD with beige. The presented millisecond values are approximated up to the order of magnitude.

In this section, we review CABS applications to simulations of protein–peptide binding (Section 3.1) and folding of globular proteins (Section 3.2). We briefly discuss modeling results for the binding of three protein–peptide systems and protein folding of one protein system. Figure 4 shows native conformations of these systems determined by X-ray crystallography or NMR. In the figure, they are arranged according to the size of a fully flexible fragment of the modeled system, effective timescales required for a meaningful simulation of their motions, and thus the modeling difficulty: (1) modeling of FxxLF motif peptide docking to an androgen receptor (AR), (2) investigation of binding and folding of an unstructured pKID protein to KIX protein, (3) modeling of p53-derived peptide docking to the MDM2 protein receptor with partially unstructured regions, and (4) simulation of the de novo folding of barnase. The simulations were performed using the CABS-dock method for protein–peptide docking [62] and CABS-flex methodology [72,73] that enable running de novo folding simulations.

### 3.1. Protein–Peptide Binding

**Figure 5:**
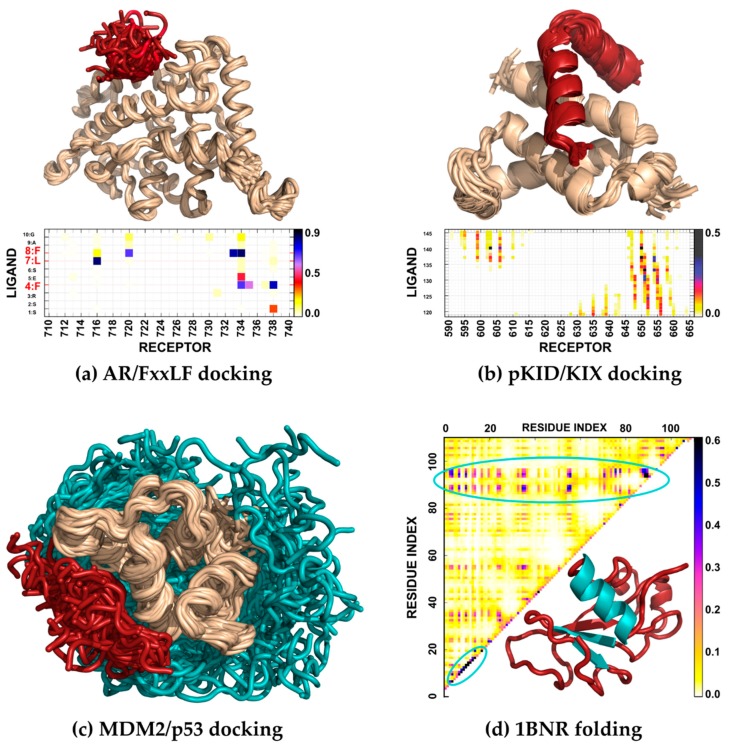
Case studies of modeling disordered or unfolded structures of proteins with CABS-based tools. In the figures, red or cyan marks structure fragments simulated as fully flexible (cyan was used to mark regions of interest discussed in the text), while beige marks regions whose motions were confined to small backbone movements (around 1 Å from the input structure). (**a**) Modeling of the dynamics of a flexible peptide representing the FxxLF motif in the proximity of the binding site of AR protein together with an averaged contact map showing frequency of residue–residue contacts during the docking simulation. (**b**) Modeling of coupled folding and binding of the disordered pKID to the KIX domain [63]; the map presents the frequency of contacts of near-native conformations obtained in the simulation. (**c**) Modeling of p53 peptide binding to the MDM2 receptor [64], which includes fully-flexible regions of the protein receptor (shown in cyan) interacting with a fully-flexible peptide (shown in red). (**d**) Modeling of barnase folding [52] in the de novo fashion (using no knowledge about the structure); the map is a residue–residue contact map showing relative contact frequencies in denaturing conditions; the protein fragments that form the folding nucleation site are colored in cyan in the presented folded structure of barnase.

The CABS-dock method has been extensively tested using the PeptiDB benchmark set of protein–peptide complexes [62,65,87]. One of the benchmark cases is the androgen receptor ligand binding domain (AR) in complex with a peptide with the FxxLF motif [88] (PDB code: 1T7R). To further analyze the interaction details of this complex, we performed blind global docking (using no knowledge about the binding site and peptide conformation) using CABS-dock [62]. As the input we used information on peptide sequence (incorporating the FxxLF motif: SSRFESLFAGEKESR), peptide secondary structure information assigned by the DSSP method [89] and the structure of the AR protein receptor. In this docking study, the peptide structure was simulated as fully flexible, while fluctuations of the protein receptor were limited to small backbone movements around the input structure (around 1 Å). The docking simulation started from random peptide conformations placed in random positions around the receptor structure. During simulation, the peptide remained unstructured until it was bound to the receptor binding site (Figure 5a). The docking simulations provided a set of high-quality models—the best model was characterized by a peptide-RMSD (root-mean-square deviation) value of 1.97 Å—and contact maps in strong agreement with the experimental data. As expected from the experimentally obtained structures and sequence analysis [88] the FxxLF interaction motif residues were most frequently involved in stabilizing hydrophobic interactions with the receptor. These high-frequency contacts are clearly visible in Figure 5a. 

The study of the pKID/KIX system [63] involved performing a folding simulation of an intrinsically disordered protein (pKID) and its binding to a well-structured KIX receptor (Figure 5b). According to the experimental studies, the pKID structure is disordered in its unbound form with a slight propensity toward a helix (for detailed description on how one-dimensional secondary structure information is used in the CABS model see [78]). In the complex with the KIX protein, pKID adopts a characteristic conformation of two perpendicular helices that wrap around the receptor. However, most simulation results for the coupled folding and binding of this system published prior to the CABS-based study used models which biased pKID toward its native conformation (see the discussion in [63]). Using our method for studying this system enabled fully flexible treatment of the pKID protein. The obtained results [63] suggested the binding mechanism that involve two encounter complexes and were in well agreement with the available NMR experimental data. The predicted models presented high fractions of native contacts and allowed identification of residues essential for the binding and stabilization of the complex. 

In the simulation of MDM2/p53 binding [64], the most challenging task was to adequately model the flexibility of the relatively long, unstructured regions of the protein receptor in addition to the fully flexible peptide [64,90] (Figure 5c). To provide a detailed insight into MDM2/p53 binding, we performed CABS-dock simulations and captured system behavior in agreement with the experimental data [64]. During the simulation, the flexible N- and C- terminal MDM2 fragments remained significantly disordered. The best resulting model was characterized by a peptide-RMSD value of 2.76 Å and 54% of the native contacts while the top ranked model by 3.74 Å and 60%, respectively. During simulations, we observed ensembles of models in which the peptide adopted different conformations loosely bound to the binding site and models in which the N-terminal highly flexible MDM2 fragment was interacting with the binding site. These findings are in agreement with the experimental data suggesting that p53-MDM2 binding is affected by significant rearrangements of the N-terminal MDM2 fragment (see discussion in [64]).

### 3.2. Folding and Flexibility of Globular Proteins

The CABS model has been applied to de novo simulations of protein folding (using no knowledge about the protein structure) for several model systems that have been extensively studied by experiment and simulation tools. Those studies include barnase [50,52], chymotrypsin inhibitor [50,52], B1 domain of protein G [49,50], B domain of protein A [53], and others [50,54]. The CABS modeling protocol was also extended to enable studies of the chaperonin effect on the folding mechanism [55]. In these works, various parameters have been studied, including residue–residue contact frequency, radius of gyration, residual secondary structure and others. The obtained pictures, which covered protein dynamics from highly denatured states to ensembles close to the folded states, agreed well with available experimental data.

For example, simulation of barnase folding resulted in the adequate reproduction of the folding pathway in strong agreement with NMR data for denatured states and phi-value analysis [52]. The performed simulations show that barnase folding starts with developing a folding nucleation site that consists of protein fragments corresponding to two strands of a beta sheet and one of the helices in the folded structure (presented in Figure 5d). In addition, the characteristic patterns of hydrophobic interactions that are crucial for the initiation and sustenance of folding are in agreement with the experimental data (see discussion in Reference [52], the contact map resulting from these simulations is presented in Figure 5d).

## 4. Conclusions

The presented case studies review the applications of the CABS model in simulations of disordered or unfolded protein states. As discussed, the method succeeded in capturing the experimentally determined features of the investigated systems, such as binding site localization, key contacts, peptide hot-spot areas, distinctive conformational states of the system, transient encounter complexes and intermediate states in protein folding [49,52,63,64]. Additionally, CABS enables an investigation of fluctuations of globular proteins around the native (input) structure [69,70,71,72,73]. 

There is a number of tools commonly used for sampling of disordered protein states, which predictions agree with the experimental studies [91,92,93,94,95]. The CABS method is complementary to these and provides a unique approach allowing for effective modeling both ordered and disordered elements of the system. As observed in many previous studies, these features of CABS method allow for providing accurate pictures of folding pathways [49,52,53,54,55,56,60] and near-native dynamics [70,71]. Obviously, due to its coarse-graining, the geometric details are missed, and their reconstructions is approximate [11,28]. The main distinctive feature of CABS method as compared to the available tools is that the ensemble generation is (pseudo-)energy driven and thus may provide some information on the dynamics on the system. This is not the case in the above-mentioned examples of methods based on random-walk [91,92,95]. 

On the other hand, CABS force field side-chain interactions escape a clear interpretation, which may be a disadvantage compared to physics-based approaches that allow for straightforward and detailed description of each of the terms [93,94].

It is, however, noteworthy that statistical force fields suffer from inherent limitations, depending on the chosen method of derivation. The most commonly discussed challenges include the transferability, solvent interactions and integration of experimental data. Here, we briefly summarize these topics, a detailed discussion of the limitations of this approach, and possible workarounds may be found in review works [11,17]. The transferability of statistical force fields may be limited as they are applicable always to a certain subset of proteins. Therefore, the performance of knowledge-based approaches may be poor for rare or atypical structures, for which appropriate statistics of contact patterns could not be collected. It should also be noted that interactions with solvent are averaged and treated implicitly, which may lead to significant discrepancies if the method is applied to non-standard solvent conditions (such as extreme pH values). The CABS force field is derived assuming averaged effect solvent conditions for folded globular proteins. Therefore, a subtle effect of small molecules, such as pH, cannot be simulated in a strict fashion, although averaged effects (see modeling the chaperonin effect [55]) can be approximately taken into considerations.

One of the most challenging tasks in modeling protein systems is the effective incorporation of sparse experimental data to drive the modeling procedure. In the CABS model, the experimental data may be readily introduced into the simulation as geometry distance restraints and weighted according to their certainty. A thorough discussion of this possibility is presented in the documentation of CABS-based tools for the fast modeling of protein flexibility and protein–peptide docking [66,72,73]. On a similar basis, CABS simulations can be guided by computational predictions from other sources or integrated with other modeling tools of various resolution. Therefore, the CABS model can be incorporated into integrative modeling pipelines that would benefit from its effective sampling scheme. The recently published standalone application and web server tools are available for integration with external pipelines (access links are presented in the last paragraph of Section 2).

## Abbreviations

CABS	Cα, Cβ, Side chain model
MC	Monte Carlo
NMR	nuclear magnetic resonance
MD	molecular dynamics
CG	coarse-grained
AR	androgen receptor
DSSP	dictionary of protein secondary structure
RMSD	root-mean-square deviation of atomic positions
PDB	Protein Data Bank
CASP	Critical Assessment of protein Structure Prediction
